# The Evolution and Future of Nursing Education: Bridging Evidence‐Based Practice and Hybrid Intelligence

**DOI:** 10.1111/jan.70434

**Published:** 2025-12-02

**Authors:** Kristina Mikkonen

**Affiliations:** ^1^ Research Unit of Health Sciences and Technology, Faculty of Medicine University of Oulu Oulu Finland; ^2^ Medical Research Center Oulu University of Oulu and Oulu University Hospital Oulu Finland; ^3^ MRC Department of Nursing Midwifery and Health, Faculty of Health and Life Sciences, Northumbria University Newcastle upon Tyne UK; ^4^ Department of Evidence‐Based Clinical Nursing, Division of Health Sciences Graduate School of Medicine, University of Osaka Osaka Japan

## 1 Introduction

Over the past five decades, nursing education has undergone a profound transformation that mirrors the evolving complexity of healthcare systems, the diversification of patient populations, and the rapid advancement of science and technology. From its early vocational roots to becoming an evidence‐driven, research‐informed, and globally networked discipline, nursing education has been a cornerstone in shaping both professional identity and healthcare delivery (Meleis 2018; International Council of Nurses [ICN] 2021). The continuous interplay between education, practice, and research has not only elevated the status of nursing within the healthcare hierarchy but also expanded its scope of practice, professional autonomy, and contribution to interdisciplinary collaboration (Benner et al. 2010).

The 1970s marked the beginning of a paradigm shift in nursing education as academic institutions began integrating evidence‐based principles and formalized curricula to strengthen the scientific foundation of nursing practice (Melnyk and Fineout‐Overholt 2023). Over subsequent decades, the rise of advanced practice roles, the expansion of graduate education, and the integration of emerging technologies have further solidified nursing as both an art and a science (Bryant‐Lukosius and DiCenso 2004; Duchscher 2008). Each phase in this evolution has redefined how nurses learn, think, and engage with patients—transforming bedside care into an arena of analytical reasoning, cultural understanding, and ethical judgment (Leininger 2002; Watson 2008).

Today, nursing education operates at the intersection of global health challenges, technological acceleration, and societal change. It is also deeply influenced by the political and economic contexts that shape healthcare systems and education policies. Political instability, global migration flows, and widening inequalities challenge how health professionals are educated and deployed across regions (WHO 2020). The increasing demand for culturally competent care, the integration of artificial intelligence and simulation in learning environments, and the emphasis on interprofessional collaboration all reflect how education prepares nurses for a future that is dynamic and uncertain (Jeffries 2021). Aligned with the United Nations Sustainable Development Goals (particularly SDG 3 on good health and well‐being, SDG 4 on quality education, and SDG 10 on reduced inequalities), nursing education now carries a sustainability mandate—to promote equitable access to care, ethical resource use, and the development of resilient health systems capable of serving diverse populations (United Nations 2015; WHO 2021).

As the profession faces demographic shifts, workforce shortages, and new ethical dilemmas in digital healthcare, the role of education becomes more critical than ever in fostering resilience, adaptability, and lifelong learning (Frenk et al. 2010; Salminen et al. 2010). Moreover, preparing nurses to address the consequences of migration, climate change, and socio‐political polarisation requires educational frameworks that emphasise sustainability, global citizenship, and advocacy.

This paper examines the evolution of nursing education over the past 50 years, identifying key milestones, innovations, and theoretical developments that have shaped nursing as a profession. It also explores the emerging challenges that the next 50 years are likely to bring—from ageing populations and workforce shortages to technological disruption and global health crises. Finally, it considers strategic directions for nursing education and policy to ensure that the profession remains responsive, humane, and future‐ready. Through this dual lens of reflection and foresight, the paper highlights how the transformation of nursing education continues to shape not only the profession itself but also the quality, equity, and sustainability of healthcare worldwide.

## 2 Historical Overview of Nursing Education (Past 50 Years)

### 2.1 Key Developments and Milestones

**FIGURE 1:**
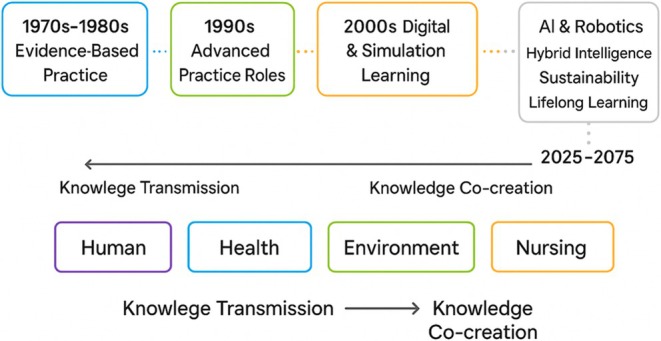
Evolution of nursing education.

The evolution of nursing education over the past half‐century reflects the profession's dynamic response to changes in health care, society, and global policy (see Figure 1). The shift from vocational training to academic, evidence‐informed education has transformed nursing into a knowledge‐based, analytical, and ethically grounded discipline (Benner et al. 2010; Meleis 2018). This evolution builds on a legacy that began more than a century earlier with the pioneering work of Florence Nightingale, whose reforms during the Crimean War continue to underpin modern nursing philosophy and pedagogy. During the Crimean War (1853–1856), Florence Nightingale revolutionised military health care through a structured approach centred on sanitation, professional discipline, and education. Recognising that infection and poor hygiene were responsible for most wartime deaths, she introduced rigorous protocols for cleanliness, ventilation, and waste management, which drastically reduced mortality rates (Dossey 1998). Her innovative use of data and statistical analysis revealed that preventable diseases, not battle injuries, accounted for the majority of fatalities. This insight prompted the British government to reform military health care systems.

Nightingale also restructured ward management by organising nursing shifts, standardising care procedures, and emphasising both physical and psychological well‐being. She ensured that patients received nutritious food, clean bedding, and even opportunities for recreation to promote recovery and morale. In parallel, she trained her nurses systematically, laying the foundation for structured professional education and establishing nursing as a respected vocation rooted in science, ethics, and compassion.

This strategic and analytical approach to care became the template for modern nursing education. Nightingale's insistence on sanitation, observation, and systematic training can be seen as the first form of evidence‐based practice, linking empirical data with health outcomes (Dossey 1998; Melnyk and Fineout‐Overholt 2023). Her legacy remains central to the nursing ethos: a balance between rigorous scientific method and humane, patient‐centered care.

Moreover, Nightingale's holistic philosophy anticipated what later became known as the four key paradigms of nursing science—human, health, environment, and nursing. These interrelated concepts form the ontological and epistemological foundation of the discipline. The “human” dimension reflects the person as a holistic being with biological, psychological, social, and spiritual needs. “Health” is understood as a dynamic state of well‐being rather than the absence of illness. “Environment” encompasses the physical, cultural, and social contexts influencing health, while “nursing” represents the caring and relational practices that integrate knowledge, ethics, and compassion (Fawcett and DeSanto‐Madeya 2013). Together, these paradigms continue to inform nursing education and research, bridging Nightingale's early emphasis on environment and hygiene with contemporary frameworks of evidence‐based, person‐centred, and sustainable care.

### 2.2 1970s–1980s: The Shift to Evidence‐Based Practice

Nightingale's educational and ethical legacy laid the foundation for nursing as both a scientific and moral profession. Nevertheless, as healthcare systems evolved through the 20th century—with the rise of new diseases, specialisation, and the professionalisation of medical practice—nursing education was compelled to adapt. The mid‐20th century brought growing recognition that Nightingale's principles of disciplined observation and evidence‐informed care required renewal within a modern, research‐oriented framework. By the 1970s, these pressures culminated in a decisive shift towards academic nursing education and the formal adoption of evidence‐based practice.

The 1970s marked a major turning point in nursing education as it embraced a scientific and research‐driven framework. Evidence‐based practice (EBP) became integral to the curriculum, reinforcing the view that nursing decisions should rest on systematic inquiry rather than tradition or intuition (Melnyk and Fineout‐Overholt 2023). Programmes began incorporating research methods, ethics, and statistics, promoting analytical reasoning and critical thinking as professional norms.

The period also saw the consolidation of nursing within universities, marking the transition from hospital‐based apprenticeships to higher education degrees. This structural shift expanded the intellectual scope of nursing, enabling the development of independent academic departments and research agendas (Meleis 2018). Legislative reforms in countries such as the UK, the United States, and the Nordic region helped establish national councils to regulate educational standards, professional registration, and practice requirements (WHO 2020).

By the late 1980s, nurses were not only practicing evidence‐informed care but also contributing to the generation of new knowledge. This move towards a scholarly profession reinforced nursing's status as a scientific discipline and positioned educators as key actors in improving health system quality and safety (Salminen et al. 2010).

### 2.3 1990s: Emergence of Advanced Practice Nursing (APN)

The 1990s witnessed the rise of Advanced Practice Nursing (APN), a development that redefined professional boundaries and education. The creation of roles such as nurse practitioners, clinical nurse specialists, and nurse consultants responded to increasing demand for specialised, autonomous practitioners capable of managing complex clinical situations (Bryant‐Lukosius and DiCenso 2004).

This expansion required master's‐level education that integrated clinical expertise with leadership, research, and policy skills. Curricula became increasingly focused on diagnostic reasoning, advanced pharmacology, and interprofessional collaboration. Theoretical models such as Benner's “Novice to Expert” framework (1984) and Leininger's Transcultural Nursing Theory (2002) shaped these programmes, reinforcing reflective and culturally sensitive practice.

The recognition of APNs as independent professionals strengthened nursing's influence across healthcare systems. In many countries, these practitioners became vital in extending access to care, particularly in underserved and rural regions (ICN 2021). The educational frameworks developed during this period laid the groundwork for doctoral and practice‐based research degrees that continue to shape the profession today.

### 2.4 2000s: Technology Integration in Nursing Education

The early 21st century brought a wave of technological innovation that profoundly altered both the methods and philosophy of nursing education. Digital learning environments, online platforms, and simulation‐based pedagogy became mainstream as educators sought to mirror the increasingly technological nature of healthcare (Jeffries 2021).

Simulation—ranging from low‐fidelity manikins to sophisticated virtual environments—offered students safe opportunities to apply theoretical knowledge, develop teamwork, and practice clinical judgment under realistic conditions. Research has shown that simulation‐based learning enhances confidence and reduces clinical errors (Jeffries 2021; Ropponen et al. 2025).

The integration of e‐learning and blended delivery models made nursing education more flexible and inclusive, particularly for working professionals and learners in remote areas. Programmes increasingly incorporated training in digital health, telemedicine, and electronic health records, recognising that data management and digital literacy were becoming essential clinical skills (Frenk et al. 2010).

These developments not only expanded access to education but also contributed to a global nursing identity that values continuous learning and adaptation to technological change (WHO 2021).

### 2.5 2010s–Present: Interdisciplinary, Global, and Digitally Transformed Education

In the past decade, nursing education has evolved towards a more interdisciplinary, global, and sustainability‐oriented paradigm. Programmes now embed interprofessional education as a core component, enabling nurses, physicians, and allied health professionals to train collaboratively for integrated patient care (Frenk et al. 2010).

Global health literacy and cultural competence have also gained prominence. Building on Leininger's transcultural foundations, educators have expanded curricula to include themes such as migration, climate change, and health equity. These priorities align with the United Nations Sustainable Development Goals (SDGs)—particularly SDG 3 (Good Health and Well‐being), SDG 4 (Quality Education), and SDG 10 (Reduced Inequalities)—which position nursing education within a broader framework of social responsibility, global citizenship, and sustainability (United Nations 2015; WHO 2021).

However, the COVID‐19 pandemic (2020–2022) represented a defining inflection point. The abrupt suspension of in‐person learning and clinical placements forced nursing schools globally to adopt remote and hybrid learning modalities almost overnight. Educators rapidly integrated digital platforms, virtual simulations, and telehealth training into curricula to sustain learning continuity (Dewart et al. 2020; Morin 2020). This accelerated digitalisation not only mitigated the disruption of traditional teaching but also permanently reshaped pedagogical practices and attitudes towards technology in nursing education (Mikkonen et al. 2024, 2025; Petäistö et al. 2025).

Simulation‐based and immersive learning tools—including virtual reality (VR), augmented reality (AR), and artificial intelligence (AI)—matured rapidly during this period, evolving from supplementary aids into core educational instruments (Kovalainen et al. 2025; Ropponen et al. 2025). AI‐driven virtual patients, robotic simulators, and data‐informed adaptive learning systems began offering personalised learning experiences and continuous performance feedback (Harmon et al. 2021). These innovations extend beyond skill acquisition; they model the human–machine collaboration that increasingly defines modern healthcare environments. Nurses must now navigate care systems where digital monitoring, predictive analytics, and automation operate alongside empathic, person‐centred care (Badawy et al. 2025).

This hybrid intelligence paradigm (Järvelä et al. 2023)—the integration of human reasoning and machine intelligence—represents a philosophical evolution in nursing education (Mikkonen et al. 2025). It challenges traditional pedagogies by demanding new competences: data literacy, digital ethics, systems thinking, and algorithmic accountability (WHO 2023). Importantly, it also reaffirms nursing's humanistic core. As automation expands, education must continue cultivating empathy, moral reasoning, and cultural awareness to ensure technology enhances rather than replaces compassionate care.

The integration of digitalisation and hybrid intelligence thus marks not merely a technological shift but a transformation in epistemology—a redefinition of what it means to be knowledgeable, competent, and human in an AI‐augmented healthcare landscape. This transition prepares future nurses to lead ethically, think critically, and care collaboratively in a world where data, diversity, and compassion must coexist.

### 2.6 Impact on the Nursing Profession and Patient Care

Across the past 50 years, the cumulative impact of these developments has been profound. Nursing education has elevated the profession from a technical vocation to a scientific and policy‐influencing discipline. Evidence‐based practice and advanced education have demonstrably improved patient outcomes, safety standards, and healthcare efficiency (Bryant‐Lukosius and DiCenso 2004; Melnyk and Fineout‐Overholt 2023).

The introduction of simulation and interprofessional learning has enhanced teamwork, communication, and leadership skills, while the globalisation of curricula has expanded nurses' ability to deliver culturally responsive care. Furthermore, the growing presence of nurses in research, academia, and policy‐making underscores the profession's expanding influence on health system design and governance (ICN 2021; WHO 2021).

Yet, through all these advancements, the ethos established by Florence Nightingale endures. Her principles of sanitation, education, and compassionate service continue to underpin modern nursing pedagogy. In this sense, the historical evolution of nursing education is both a continuum of Nightingale's vision and a reflection of contemporary challenges—technological, ethical, and humanitarian—that shape the global healthcare landscape today.

## 3 Influential Research, Policies, and Technological Advancements

### 3.1 Pivotal Research and Theoretical Contributions

The development of nursing education has been guided by a series of influential research programmes and theoretical models that have redefined how nurses understand, deliver, and evaluate care. Since the mid‐20th century, nursing scholars have progressively established a body of disciplinary knowledge that integrates theory, research, and practice, thereby elevating nursing from a vocational craft to a scientific profession (Fawcett and DeSanto‐Madeya 2013; Meleis 2018).

Among the most transformative theoretical frameworks is Patricia Benner's “Novice to Expert” model (1984), which conceptualized nursing competence as a developmental process shaped by experiential learning. This model reframed education by emphasizing reflective practice, critical thinking, and clinical reasoning as essential components of professional growth (Benner et al. 2010). Similarly, Jean Watson's Theory of Human Caring (2008) positioned caring as the moral and epistemological core of nursing, asserting that the integration of scientific knowledge and compassionate practice defines professional excellence. Watson's work has influenced both curricula and pedagogical approaches that stress empathy, relational ethics, and patient‐centered care.

Madeleine Leininger's Transcultural Nursing Theory (2002) introduced the concept of cultural competence long before globalization and migration brought diversity to the forefront of healthcare education. Her emphasis on cultural understanding as an element of quality care has inspired educational reforms that embed global health and equity perspectives into nursing curricula (Salminen et al. 2010). In a similar vein, Dorothea Orem's Self‐Care Deficit Theory (1991) and Sister Callista Roy's Adaptation Model (1984) have informed the teaching of holistic assessment and patient empowerment, reinforcing the view of patients as active participants in health rather than passive recipients of care.

In recent years, interprofessional education and simulation‐based learning research have emerged as key areas of scholarship. Pioneers such as Pamela Jeffries (2021) developed simulation theory to formalize experiential learning in controlled environments, demonstrating how reflective debriefing and scenario‐based training enhance clinical judgment. More recent studies (Kovalainen et al. 2025; Ropponen et al. 2025) have extended this work into digital and AI‐supported learning contexts, where virtual patients and adaptive technologies enable continuous assessment and personalized learning experiences.

Collectively, these theoretical and empirical contributions have shaped a multidimensional understanding of nursing knowledge—one that balances the humanistic, empirical, ethical, and aesthetic domains of care (Carper 1978; Fawcett and DeSanto‐Madeya 2013). They continue to influence contemporary education by integrating evidence, empathy, and critical inquiry within both classroom and clinical settings.

### 3.2 Policy Changes Impacting Nursing Education

Parallel to research and theory, the policy environment has profoundly influenced nursing education worldwide. The late 20th century saw the consolidation of nursing as a regulated profession, with governments and international organisations introducing universal standards for curricula, licensing, and competency assessment (WHO 2020).

In many countries, nursing education transitioned from hospital‐based diploma systems to university‐level degree programmes, a move supported by the World Health Organization and the International Council of Nurses (ICN). These reforms aimed to align nursing with international academic standards and enhance professional mobility (ICN 2021). For example, the European Union's Directive 2005/36/EC on professional qualifications established minimum training hours and core competencies for nursing graduates across member states, promoting harmonisation and workforce flexibility (European Commission 2005).

Licensing and accreditation reforms have also been pivotal in ensuring accountability and public safety. National regulatory bodies, such as the Nursing and Midwifery Council (UK), the American Association of Colleges of Nursing (AACN), and similar institutions across Asia and the Nordic region, have introduced outcome‐based education frameworks emphasizing competence, ethics, and lifelong learning (Salminen et al. 2010).

Recent policy directions have increasingly emphasized sustainability, digital competence, and workforce resilience. The WHO Global Strategic Directions for Nursing and Midwifery 2021–2025 call for investment in education, leadership, and decent work, recognizing nurses as central to achieving the United Nations Sustainable Development Goals (WHO 2021). These frameworks encourage integrating climate health, equity, and digital literacy into nursing education to prepare future professionals for complex, interconnected challenges.

The COVID‐19 pandemic further exposed systemic gaps in policy preparedness, prompting new strategies for remote education regulation, telehealth training, and international credential recognition (Morin 2020; Dewart et al. 2020). In response, universities and ministries of health implemented emergency guidelines that have since evolved into enduring policies supporting hybrid and technology‐enhanced learning. These reforms underscore that policy and pedagogy must remain adaptive to technological and societal change.

### 3.3 Technological Advancements and Innovation

Technological advancement has been one of the most powerful forces transforming nursing education and practice. The integration of electronic health records (EHRs), telehealth, and digital documentation systems into curricula reflects the growing demand for data literacy and digital professionalism (Konttila et al. 2019; Badawy et al. 2025). Nurses are now trained not only to use these tools but to interpret, manage, and ethically evaluate data to improve patient outcomes.

The rise of simulation technology has also revolutionized pedagogical methods. High‐fidelity manikins, immersive virtual environments, and AI‐driven learning systems allow students to experience realistic clinical scenarios and receive real‐time feedback (Cant and Cooper 2010; Jeffries 2021; Kovalainen et al. 2025). These innovations have shifted nursing education from a passive learning approach to an active, experiential engagement, enabling educators to assess competence in decision‐making, teamwork, and patient communication more effectively.

Artificial intelligence (AI) and robotics represent the newest frontier of innovation. AI‐driven tutoring systems, predictive analytics, and robotic assistants are being incorporated into both learning and clinical environments (Harmon et al. 2021). These tools foster human–machine collaboration, where nurses combine algorithmic insights with empathy and professional judgment to deliver high‐quality care (WHO 2023). Moreover, the emergence of hybrid intelligence models—where human expertise and machine computation operate synergistically—has significant implications for curriculum design. Education must now address digital ethics, algorithmic bias, and the emotional dimensions of technology‐mediated care.

Telehealth has similarly transformed the scope of nursing practice and education. Once considered supplementary, remote care delivery became essential during the pandemic and remains integral to post‐pandemic healthcare systems (Morin 2020). As a result, nursing students are increasingly trained to deliver compassionate, effective, and secure care across digital interfaces—a skill that combines technological proficiency with communication sensitivity.

These innovations collectively demonstrate that technology is not a replacement for human care but an extension of it. The ongoing challenge for educators lies in maintaining the balance between technical competence and the enduring humanistic essence of nursing—a challenge first articulated by Nightingale and now reimagined in the era of digital and hybrid intelligence.

## 4 Challenges Facing the Nursing Profession in the Next 50 Years

As nursing enters the mid‐21st century, the profession faces an increasingly complex set of challenges shaped by demographic shifts, technological transformation, and global health instability. The next 50 years will test nursing's adaptability, ethical foundations, and educational capacity to prepare practitioners for rapidly evolving healthcare systems. These challenges are interlinked—social, technological, and ecological factors converge to redefine not only how nurses work but what it means to provide care in an interconnected, data‐driven, and uncertain world.

### 4.1 Demographic Changes

Global demographic transitions are reshaping the demand for nursing services. Populations are aging rapidly, chronic diseases are rising, and the need for long‐term, complex care is growing (WHO 2023). In Europe, North America, and much of East Asia, the proportion of people aged 65 and over will nearly double by 2050, intensifying the pressure on health and social‐care systems (United Nations 2022).

This demographic shift will magnify the workforce shortage already evident in many regions. The WHO estimates a global deficit of almost 10 million nurses by 2030 (WHO 2021). Unless addressed through strategic recruitment, international mobility, and improved retention, this shortage will threaten access to equitable care.

At the same time, populations are becoming more culturally and linguistically diverse due to migration and global mobility (Joensuu et al. 2024; Kamau et al. 2022). Nurses must therefore be prepared to deliver culturally competent, person‐centred care that recognises differences in language, health beliefs, and social contexts (Salminen et al. 2010). Educational programmes will need to strengthen intercultural communication and ethical reasoning, enabling nurses to serve heterogeneous populations while combating health inequities and discrimination.

The demographic evolution also demands new models of care for older adults, including home‐based monitoring, community health initiatives, and intergenerational learning. As longevity increases, so too does the need for integrated gerontological expertise that combines medical, psychological, and social dimensions of care.

### 4.2 Technological Advancements and AI in Healthcare

The coming decades will see an even deeper fusion of nursing and technology. AI, robotics, and digital decision‐support systems are already altering clinical workflows, and their influence will intensify (Badawy et al. 2025; Kovalainen et al. 2025). These technologies promise enhanced diagnostic accuracy, predictive analytics for patient deterioration, and automation of routine documentation, allowing nurses to focus more on complex and relational aspects of care.

Nevertheless, such innovation also brings ethical, educational, and professional challenges. The nurse of the future must balance human‐centred care with machine assistance, maintaining empathy, presence, and moral judgment in environments saturated with data. Training programmes will need to cultivate digital literacy, critical appraisal of algorithms, and awareness of bias and privacy concerns (World Health Organization (WHO) 2023).

The emergence of robotic assistants in elder care and rehabilitation settings illustrates both potential and tension. Robots can reduce physical strain and support repetitive tasks, but they also raise questions about relational authenticity and patient dignity. Nursing ethics must therefore evolve to encompass the “human–machine interface”, defining professional boundaries and accountability when technology participates in caregiving (Stahl and Coeckelbergh 2016).

Moreover, the integration of hybrid intelligence systems—where human expertise and AI interact dynamically—will require rethinking educational paradigms (Järvelä et al. 2023). Simulation, virtual reality, and adaptive AI tutors will become standard in nursing schools, offering immersive and personalized learning (Mikkonen et al. 2025). However, without careful pedagogical design, over‐reliance on automation may erode the reflective and interpersonal capacities central to nursing identity.

To remain future‐ready, nursing must adopt a dual approach: embracing technological advancement while safeguarding the human essence of care. Curricula should embed digital ethics, systems thinking, and resilience training, preparing graduates to lead ethically within AI‐enhanced healthcare.

### 4.3 Global Health Trends and Public Health Threats

The experience of the COVID‐19 pandemic demonstrated the centrality of nurses in crisis response, surveillance, and recovery (Dewart et al. 2020). Future pandemics, climate‐related disasters, and geopolitical conflicts are likely to increase in frequency, demanding a resilient, globally coordinated nursing workforce. Education must therefore emphasize outbreak preparedness, infection control, and adaptive leadership under uncertainty.

Globalisation has also heightened awareness of planetary health—the recognition that human well‐being depends on ecological stability. Climate change is already driving new disease patterns, displacement, and mental‐health stressors. Nurses will play critical roles in mitigation and adaptation strategies, from community education on sustainable lifestyles to advocacy for environmentally responsible healthcare systems (Evans‐Agnew et al. 2024).

The next half‐century will also require renewed focus on mental health and preventive care. Post‐pandemic societies face rising levels of anxiety, burnout, and loneliness. Nurses, often serving as the first point of contact in communities, must be equipped with skills in psychological first aid, counselling, and digital mental‐health interventions (ICN 2022). Integrating these competences into curricula will ensure that mental health is viewed not as a specialist niche but as a universal component of holistic care.

Emerging global health threats—antimicrobial resistance, food insecurity, and population displacement—will further expand nursing's public‐health responsibilities. Collaboration with international agencies, interdisciplinary collaboration, data sharing, and global research networks will be crucial. The nursing profession's advocacy capacity must therefore grow alongside its clinical expertise, enabling nurses to influence policy at local and international levels.

### 4.4 Looking Forward

The coming 50 years will challenge nursing to maintain its humanistic foundation amid accelerating technological and societal change. The profession must cultivate adaptability, ethical leadership, and intersectoral collaboration. Investment in lifelong learning, research capacity, and international partnerships will be essential to sustain innovation and resilience.

Ultimately, the greatest challenge may lie in preserving the equilibrium between science and compassion—between the precision of data and the unpredictability of the human condition. Nursing's enduring strength will continue to rest on its ability to combine evidence, empathy, and advocacy in pursuit of equitable, sustainable, and humane healthcare for all.

## 5 Strategies for Addressing Future Challenges

The next half‐century will require nursing to sustain its humanistic roots while innovating in response to global, demographic, and technological transformation. The complexity of emerging healthcare systems demands that nursing education, policy, and professional culture become more adaptive, collaborative, and ethically grounded. The following strategies outline pathways for strengthening nursing's capacity to meet the challenges of the future.

### 5.1 Adapting Education and Curriculum

Nursing education must evolve from static, content‐based teaching to adaptive, technology‐focused, and globally relevant learning systems. As healthcare becomes increasingly digital, curricula should integrate simulation, data analytics, AI, and telehealth not merely as tools, but as conceptual frameworks for understanding care in hybrid environments (WHO 2023).

Adaptive curricula should prioritise competency‐based education—emphasising mastery of practical and cognitive skills through iterative feedback and simulation‐based assessments (Jeffries 2021). This approach aligns with the WHO's call to embed digital literacy, systems thinking, and sustainability within health education to prepare the workforce for future crises (WHO 2021).

Equally critical is the inclusion of global health competences. The challenges of migration, pandemics, and climate change underscore the need for culturally intelligent and globally minded practitioners (United Nations 2015; Salminen et al. 2010). Interprofessional education models can be expanded to include cross‐border collaboration, encouraging students to work virtually with peers in other countries on shared projects addressing global health issues (Mikkonen et al. 2025). Such exposure enhances mutual understanding, ethical reasoning, and resilience—competences essential for leading in multicultural and crisis‐prone contexts.

Resilience training must also become integral to nursing education. The COVID‐19 pandemic revealed the psychological vulnerability of health workers and the necessity of emotional intelligence, self‐care, and moral resilience in maintaining professional longevity (Dewart et al. 2020; ICN 2022). Embedding reflective practice, mindfulness, and peer‐support frameworks into curricula can cultivate adaptability, ensuring that nurses remain both clinically competent and psychologically robust.

Ultimately, adapting education requires a paradigm shift—from transmission of knowledge to co‐creation of wisdom. Educators and students alike must act as partners in inquiry, innovation, and reflection. This dynamic approach keeps nursing education relevant to rapidly shifting technologies and evolving societal needs.

### 5.2 Policy and Advocacy for the Nursing Profession

Strategic policy action is essential to ensure that nurses can thrive in the changing landscape of global healthcare. Governments, professional associations, and academic institutions must collaborate to implement policies that recognize nurses as central to the resilience and innovation of the health system.

Key priorities include addressing workforce shortages and retention through fair remuneration, safe staffing levels, and career development opportunities (Niskala et al. 2020). Policy frameworks should also support lifelong learning and credential mobility, enabling nurses to update competencies across borders and career stages (WHO 2021).

Advocacy for decent working conditions—including manageable workloads, psychological safety, and protection against workplace violence—is central to sustaining motivation and quality of care (ICN 2022). As technology reshapes practice, regulations must evolve to define scope, accountability, and ethical boundaries in AI‐assisted and remote care environments (Badawy et al. 2025).

In education policy, governments should promote investment in simulation centres, digital infrastructure, and research capacity within nursing schools. Collaboration between universities, industry, and health authorities can accelerate the development of innovative learning ecosystems that reflect real‐world challenges.

Nurses must also strengthen their presence in policy‐making and governance. Representation on hospital boards, national health councils, and global advisory panels ensures that clinical insight informs strategic decision‐making. The profession's advocacy must extend to climate action, human rights, and sustainable health policies, reflecting the interconnectedness of health and planetary well‐being (Evans‐Agnew et al. 2024).

The WHO Global Strategic Directions for Nursing and Midwifery (2021–2025) call for building leadership pipelines and fostering political competence among nurses. Leadership education—at both undergraduate and postgraduate levels—should thus include public policy, health economics, and systems management, equipping nurses to influence decisions rather than merely implement them.

### 5.3 Fostering a Culture of Innovation and Lifelong Learning

The pace of healthcare change demands a professional culture rooted in innovation, curiosity, and continuous learning. Innovation in nursing practice does not solely refer to technological invention but also to creative problem‐solving, service redesign, and collaborative inquiry.

Encouraging innovation begins with cultivating psychological safety—environments where nurses feel empowered to question practices, propose improvements, and learn from mistakes without fear of reprisal (Edmondson 2018). Academic–clinical partnerships can accelerate this by linking frontline experience with research and innovation hubs, allowing practitioners to co‐design solutions for patient and organizational challenges.

Mentorship and coaching remain vital mechanisms for transmitting tacit knowledge and professional values. Structured mentorship programmes—both within workplaces and through digital platforms—can enhance retention, promote leadership development, and reduce burnout (Benner et al. 2010). Mentorship should also extend internationally, fostering networks that enable nurses to exchange expertise across cultural and disciplinary boundaries (Herinek et al. 2025).

Continuous professional development must evolve into lifelong, flexible learning ecosystems. Micro‐credentialing, modular courses, and AI‐driven personalized learning paths can help nurses continually update their skills while balancing professional and personal responsibilities (Harmon et al. 2021). This model reflects the future of hybrid learning and supports workforce agility.

Research engagement is another pillar of innovation. Encouraging nurses to participate in or lead research projects strengthens evidence‐based practice and promotes leadership visibility within the scientific community (Fawcett and DeSanto‐Madeya 2013). Academic institutions should provide mentorship and funding structures that nurture early‐career nurse researchers and recognize practice‐based innovation as a scholarly contribution.

Cultivating innovation ultimately requires aligning the four key paradigms of nursing—human, health, environment, and nursing—with emerging realities of digitalisation and sustainability. As hybrid intelligence and environmental change reshape healthcare, the profession must continue to unite science and empathy, technological skill and moral wisdom, individual expertise and collective learning.

## 6 Conclusion

The evolution of nursing education over the past 50 years reflects one of the most profound transformations in the history of healthcare. From Florence Nightingale's 19th‐century reforms—anchored in sanitation, systematic observation, and moral duty—to the research‐driven, technology‐enabled, and globally connected discipline of today, nursing has continually adapted to the changing needs of societies and patients. The profession's journey from hospital‐based apprenticeship to higher education and scholarly inquiry has elevated its intellectual and ethical stature, solidifying its role as a cornerstone of modern healthcare systems.

Throughout this evolution, key developments have shaped nursing's identity. The integration of evidence‐based practice in the 1970s and 1980s grounded nursing in scientific rigor. The emergence of advanced practice roles in the 1990s expanded professional autonomy and improved access to specialized care. The digital and simulation revolutions of the 2000s transformed teaching and learning, fostering more interactive and experiential models. The 2010s onward have brought a new emphasis on interdisciplinary, global, and sustainability‐oriented education, preparing nurses to navigate interconnected health, social, and environmental challenges.

These milestones have collectively redefined the profession's contribution to patient care, research, and policy. Nursing education has not only enhanced clinical competence but also cultivated critical thinkers, innovators, and advocates capable of shaping the future of health systems. Yet, as the world enters an era of demographic shifts, technological acceleration, and geopolitical uncertainty, nursing faces unprecedented demands. The growing complexity of care environments requires professionals who are not only clinically proficient but also digitally literate, culturally intelligent, and resilient.

The readiness of the profession for these challenges depends on its ability to sustain adaptive education, evidence‐informed policy, and a culture of lifelong learning. Future curricula must merge scientific knowledge with emotional intelligence, ensuring that empathy and ethics remain central to technologically mediated care. Policy frameworks must protect and empower the workforce—investing in leadership development, decent working conditions, and interprofessional collaboration. The profession must also embrace its global responsibility: addressing inequities, advocating for sustainable health, and contributing to planetary well‐being.

Looking ahead, the greatest opportunity for nursing lies in shaping the dialogue between human compassion and machine intelligence, ensuring that technology serves as a bridge rather than a barrier to humane care. Nurses, as connectors between science and society, are uniquely positioned to guide this balance.

The call to action is clear: nursing must continue to innovate, adapt, and advocate. By investing in education that values both critical inquiry and compassion, by influencing policy grounded in justice and sustainability, and by nurturing creativity and courage in every practitioner, the profession can remain at the forefront of global health transformation. The next 50 years will demand not only technical skill but moral imagination—the capacity to envision and create a future where care, in all its forms, remains profoundly human.

## Funding

The author has nothing to report.

## Conflicts of Interest

The author declares no conflicts of interest.

## Data Availability

Data sharing not applicable to this article as no datasets were generated or analysed during the current study.
